# Patient satisfaction following Goldilocks mastectomy in non-obese women: A pilot study

**DOI:** 10.1016/j.breast.2026.104857

**Published:** 2026-06-29

**Authors:** T.K. Muntslag, M.M. van Veen, V.C. van Aalst, I.L. Holt – Kedde

**Affiliations:** University of Groningen, University Medical Center Groningen, Department of Plastic Surgery, Groningen, the Netherlands

**Keywords:** Goldilocks mastectomy, Mastectomy, Breast reconstruction, Autologous reconstruction, BREAST-Q, Oncoplastic surgery, Quality of life (QoL)

## Abstract

The Goldilocks mastectomy was described as a reconstructive option for obese patients undergoing mastectomy, aiming to reduce surgical burden by avoiding extremes of traditional reconstruction. In non-obese patients, a limitation of this technique is insufficient postoperative breast volume; however, data on patient-reported satisfaction remains scarce. In this pilot study, we evaluated postoperative satisfaction in non-obese patients undergoing Goldilocks mastectomy using the BREAST-Q questionnaire. Despite limited breast volume, patient-reported outcomes demonstrated favorable satisfaction and quality-of-life domains. These findings suggest Goldilocks mastectomy may represent a viable reconstructive option for selected non-obese patients who prefer a simpler, fully autologous approach with good outcomes.

## Graphical abstract

**Figure:**
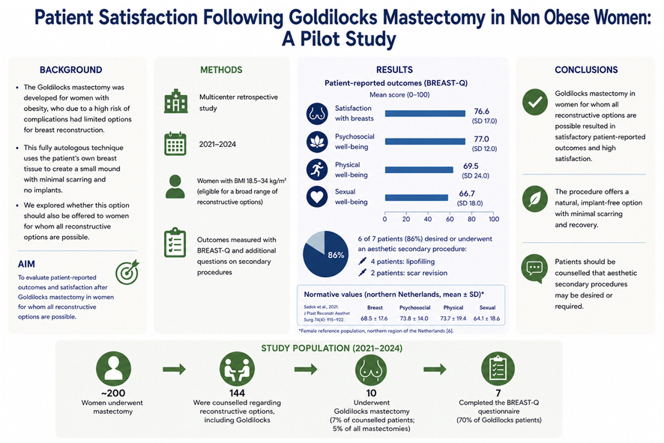

## 1 Introduction

Following mastectomy, breast reconstruction options range from alloplastic procedures using tissue expanders or implants to autologous reconstruction with a free-flap or lipofilling. Each reconstructive option is associated with distinct risks, recovery profiles, and aesthetic outcomes [1].

The Goldilocks mastectomy, first described in 2012 by Richardson and Ma, was introduced as a reconstruction alternative for obese patients aiming to limit surgical burden, by avoiding the extremes of traditional implant-based or complex autologous reconstruction [2]. The technique utilizes de-epithelialized tissue of a skin sparing mastectomy to create an autologous breast mound, thereby eliminating the immediate need for prosthetic implants or microsurgical flaps [2]. Subsequently, its use has been reported primarily in patients with macromastia or significant comorbidities who are considered at increased risk for postoperative complications [3,4].

Available outcome and satisfaction data largely reflect these high-risk patient populations, in whom the Goldilocks mastectomy is often selected due to contraindications for other reconstructive options. Consequently, patient-reported outcomes for women who are otherwise eligible for a broad range of reconstructive procedures remain insufficiently characterized. This represents an important knowledge gap, as these patients may elect a Goldilocks mastectomy for its simplicity and autologous nature rather than medical necessity.

This pilot study therefore aimed to evaluate patient-reported satisfaction and outcomes following Goldilocks mastectomy in women eligible for multiple reconstructive options, using the validated BREAST-Q instrument [1].

## 2 Methods

This retrospective, multicenter pilot cohort study included women aged ≥18 years who underwent a Goldilocks mastectomy at the University Medical Center Groningen (UMCG), Ommelander Ziekenhuis Groningen (OZG), or Treant Hospital between 2021 and 2024. Eligible patients had a body mass index (BMI) between 18.5 and 34 kg/m^2^ at the time of surgery, a range within which patients are eligible for a broad range of reconstructive options, rather than having limited options due to BMI restrictions. Patients with prior implant-based or autologous breast reconstruction applied to the breast(s) undergoing Goldilocks mastectomy were excluded. During the study period, approximately 200 women underwent mastectomy at the participating centers, of whom 144 were evaluated in the plastic surgery outpatient clinic and counselled regarding reconstructive options, including the Goldilocks procedure. Ten women ultimately underwent Goldilocks mastectomy, representing approximately 7% of counselled patients and 5% of all women undergoing mastectomy.

The study was approved by the Medical Ethics Review Committee of UMCG on 17 September 2024 (approval number 20326), and all participants provided written informed consent. Patient demographics, oncologic characteristics, surgical details, and available follow-up data were obtained from electronic medical records. Postoperative patient-reported outcomes were assessed using the BREAST-Q Reconstruction Module, a validated instrument evaluating satisfaction with breasts, psychosocial well-being, physical well-being, and sexual well-being [1]. Participants were additionally asked whether they had undergone or desired secondary procedures to improve breast appearance.

**Fig. 1:**
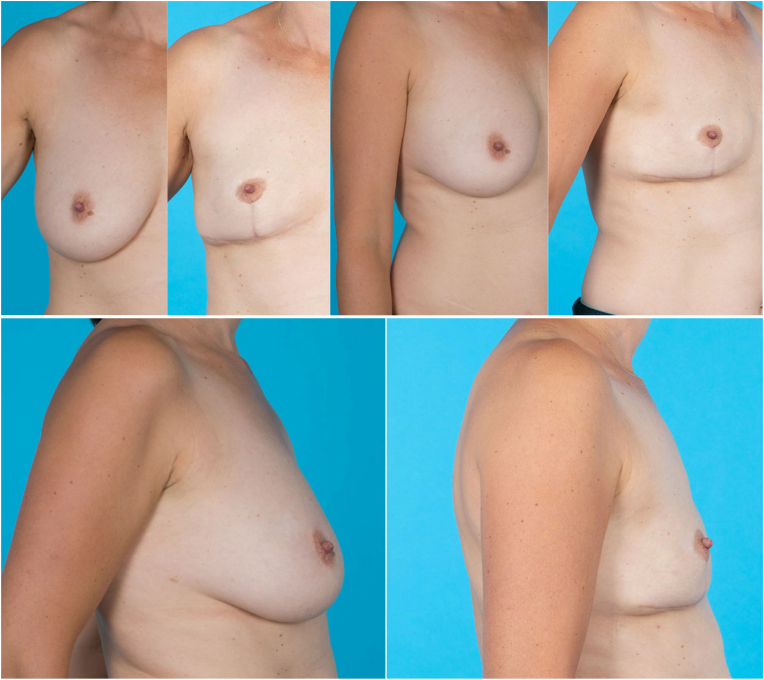
Pre- and postoperative images of 48 year-old patient with BMI 23.4 Top left: ventral view pre- (left) and postoperatively (right) Top right: Anterolateral view pre- (left) and postoperatively (right) Below: lateral view pre- (left) and postoperatively.

The Goldilocks mastectomy was performed using a skin-sparing approach with de-epithelialization of the spared skin flaps, which were folded to create an autologous breast mound without the use of implants or free-flap reconstruction (see Fig. 1). Descriptive statistics were used to summarize patient and clinical characteristics. BREAST-Q domain scores were calculated according to standard guidelines and reported as means with standard deviations. Given the exploratory nature and limited sample size of this pilot study, outcomes were compared descriptively with published normative BREAST-Q values [5,6], without formal hypothesis testing.

## 3 Results

Of the 10 patients who met the inclusion criteria, 7 (70%) completed the BREAST-Q survey and were included in the analysis. Patient demographics and BREAST-Q outcomes are summarized in Table 1. The mean age was 49 years, and the mean body mass index was 25.6 kg/m^2^. Two patients underwent unilateral Goldilocks mastectomy, while five patients received bilateral procedures.

**Table 1:** Demographics and BREAST-Q outcomes.

Demographics								Results postoperative BREAST-Q		
Patient	Age	BMI	Ptosis? Y/N Grade	Breast cancer = 1 Preventive = 0	Breasts treated with Goldilocks	Complication Y/N	Psychosocial wellbeing	Sexual wellbeing	Physical wellbeing	Satisfaction with breasts
Mean (SD)	49(8)	25.6 (3)				2/7	66 (12)	52 (17)	71 (24)	57 (7)
1	56	23.1	N	1 + 0	2	N	71	46	85	46
2	59	23.3	Y2	0 + 0	2	N	74	50	55	52
3	48	23.4	N	0	1^#^	N	77	62	68	71
4	47	23.6	N	1	1^##^	N	62	50	32	-
5	53	25.9	Y3	0 + 0	2	Y∗∗	47	20	100	58
6	43	28.7	Y1	1 + 1	2	Y∗	52	56	100	59
7	37	31.4	Y1	0 + 0	2	N	80	79	55	58

Table notes: ^#^ contralateral LD transposition without implant, ^##^ contralateral symmetry-improving breast reduction, ∗ Seroma of both breasts, clavien dindo score I [7] ∗∗ postoperative bleeding clavien dindo score IIIb [7].

The most frequently cited reasons for choosing the Goldilocks mastectomy were the autologous nature of the reconstruction, the absence of implants, and the ability to maintain a breast mound compared with traditional mastectomy. Six out of seven patients (86%) reported that their surgical outcome met their expectations. All patients reported moderate to high overall satisfaction with their reconstructive outcome (Table 1).

For six of the seven patients (86%), BREAST-Q satisfaction with breasts scores fell within one standard deviation of published normative values reported by Sadok et al. for the northern region of the Netherlands [6]. Six patients (86%) reported having undergone or considered secondary revisions to improve aesthetic outcome, including lipofilling (n = 4) and scar revision (n = 2).

## 4 Discussion

This study evaluates patient satisfaction and outcomes following Goldilocks mastectomy in non-obese patients. Existing literature has largely emphasized physician-reported concerns regarding limited breast volume, projection and potentially sub-optimal aesthetic results due to limited residual subcutaneous tissue post-mastectomy, with relatively little attention to patient-reported outcomes [4,8]. A Japanese case series reported poor physician-assessed aesthetic outcomes in three out of five women due to a “very small” breast mound, notably, these same patients frequently reported satisfaction with their results despite this limitation [8]. Similarly, Chaudhry et al. described patient satisfaction in the context of limited projection, highlighting the importance of incorporating patient-reported outcomes alongside clinical assessments when evaluating reconstructive success [4].

In our small cohort, BREAST-Q scores fell within one standard deviation of published normative values for women undergoing other autologous or alloplastic breast reconstruction in the same region, as reported by Sadok et al. [6]. Patients most frequently valued the ability to achieve autologous reconstruction without going flat, while avoiding multistage procedures, donor-site morbidity, prolonged recovery time or the use of foreign materials [[2], [3], [4]]. Although these findings must be interpreted cautiously due to the small cohort and a broad range of outcomes, they suggest that, for selected patients, the Goldilocks mastectomy may represent an acceptable reconstructive alternative rather than solely a compromise option. Therefore, in our consultations about breast reconstruction, we consider it valuable to include and explain the option of Goldilocks mastectomy alongside other reconstructive techniques as part of shared decision-making. This perspective aligns with recent reports describing comparable patient satisfaction between Goldilocks mastectomy alone and Goldilocks mastectomy combined with direct implant placement [[9], [10], [11]].

Postoperative breast volume and the potential for secondary procedures to increase breast volume remain important considerations. In our cohort, four of seven women expressed a desire for additional lipofilling, illustrating both a recognized limitation of the technique and its potential adaptability. While small initial breast volume is frequently cited as a drawback, recent studies emphasize that the preserved skin envelope allows flexibility in the type of volume increasement. Ghanouni et al. demonstrated improved outcomes following delayed implant placement after Goldilocks mastectomy compared with standard mastectomy closures [3], while Sarrami et al. reported favorable volume restoration using high-volume fat grafting, enabling further refinement without implants or extensive alloplastic reconstruction [12].

This study has important limitations, including the small sample size, absence of a control group, and the descriptive nature of the analysis, all of which restrict generalizability and preclude definitive comparative conclusions. Nevertheless, these findings highlight the importance of incorporating patient-reported outcomes when evaluating Goldilocks mastectomy and support further prospective studies to clarify its role across different patient populations.

## 5 Conclusion

Originally developed for high-risk patients, the Goldilocks mastectomy demonstrated feasible and acceptable patient-reported outcomes in this small cohort of reconstruction-eligible women. Its autologous nature and avoidance of implants may make it a suitable option for selected patients seeking a simpler reconstructive approach: however, secondary revision procedures may be required or desired in a substantial proportion of patients and should therefore be discussed during preoperative counselling. Given the limited sample size, these preliminary findings should be interpreted cautiously, and larger comparative studies are needed to better define its role.

## Disclosure

This research did not receive any specific grant from funding agencies in the public, commercial, or not-for-profit sectors. The authors have no financial, personal, political, or academic interests to declare in relation to the content of this study. Ethical approval was obtained from the Medical Ethical Review Committee (METc 20326) and all participants provided written informed consent prior to inclusion.

## CRediT authorship contribution statement

**T.K. Muntslag:** Formal analysis, Investigation, Methodology, Visualization, Writing – original draft, Writing – review & editing. **M.M. van Veen:** Conceptualization, Methodology, Writing – review & editing. **V.C. van Aalst:** Conceptualization, Methodology, Resources, Writing – review & editing. **I.L. Holt – Kedde:** Conceptualization, Methodology, Resources, Supervision, Writing – review & editing.

## Declaration of competing interest

The authors declare that they have no known competing financial interests or personal relationships that could have appeared to influence the work reported in this paper.
